# A silent epidemic of major congenital malformations in Tigray, northern Ethiopia: hospital-based study

**DOI:** 10.1038/s41598-021-00240-7

**Published:** 2021-10-26

**Authors:** Hayelom Kebede Mekonen, Yibrah Berhe, Birhane Alem Berihu, Hale Teka, Abera Hadgu, Letekirstos Gebregziabher, Etenat Halefom Berhe, Tony Magana, Afework Mulugeta

**Affiliations:** 1 College of Health Sciences, School of Medicine, Division of Biomedical Institute, Department of Anatomy, Mekelle University, Mekelle, Ethiopia; 2 College of Health Sciences, School of Medicine, Department of Gynaecology & Obstetrics, Mekelle University, Mekelle, Ethiopia; 3 College of Health Sciences, School of Medicine, Department of Pharmacology, Mekelle University, Mekelle, Ethiopia; 4 College of Health Sciences, School of Public Health, Department of Biostatistics, Mekelle University, Mekelle, Ethiopia; 5 College of Social Sciences & Languages, Department of Geography & Environmental Studies, Mekelle University, Mekelle, Ethiopia; 6 College of Health Sciences, School of Medicine, Department of Neurosurgery, Mekelle University, Mekelle, Ethiopia; 7 College of Health Sciences, School of Public Health, Department of Nutrition and Dietetics, Mekelle University, Mekelle, Ethiopia; 8 School of Medicine, Department of Anatomy and Neurobiology, Postdoctoral Fellow at University of Maryland Baltimore, Baltimore, USA

**Keywords:** Developmental biology, Structural biology, Anatomy

## Abstract

Congenital malformations are defects of the morphogenesis of organs or body during the pregnancy period and are identifiable at pre- or postnatal. They are identified as the major cause of child mortality worldwide. There is a need to understand the prevalence of congenital malformations in Tigray and Ethiopia in general as surveillance data are lacking. Hence, this study was designed to investigate the burden of major congenital malformations in the Tigray Region, Northern Ethiopia. Hospital-based cross-sectional study was conducted to identify neonates with major congenital anomalies in the labor ward admitted at six major public hospitals of Tigray region, Ethiopia between January 2018 and 2019. All newborns/neonates delivered in all study hospitals during the study period were considered as the study population. The prevalence of major congenital anomalies and the distribution of each type of major congenital anomalies within total birth were calculated. Data on maternal, and newborn demographic characteristics was collected. Statistical analysis was done using SPSS and *p* value < 0.05 was considered significant. A total of 12,225 births and terminations were recorded in the six hospitals during the study period. Of total 12,225 births and terminations examined, 383 births had major congenital malformations and the overall prevalence of congenital malformations was 3.13% of the total births examined. Congenital anomalies (CAs) of the central nervous system specifically neural tube defects (NTDs) were the commonest anomalies in this study, found in 68.7% (263NTDs/383 CAs) of the neonates with CAs. The overall prevalence of NTDs was 2.15% (263/12,225 births) of the total births examined. Maternal factors such as women 20 years of age or younger (*p* < 0.0001) and women older than 35 years of age (*p* < 0.0001), abortion history (*p* < 0.0001), gravidity above 4 (p = 0.005), were more likely associated with an increased risk of babies with congenital anomalies. Fetal factors including gestational ages below 28 weeks (*p* < 0.0001) and above 40 weeks (*p* < 0.0001) were strongly associated with an increased risk of babies with congenital anomalies. However, these associated factors were not resulted from multivariable logistic regression analysis. Thus, the result might be affected by possible confounding factors. This study has shown a high prevalence of major congenital anomalies in the study community. Of the total congenital anomalies observed, most of neonates are affected with neural tube defects, a birth defect with well–established evidence having folic acid deficiency or insufficiency is the predominant cause of spina bifida and anencephaly. This just screams urgency to implement effective/mandatory/ programs to get all women of reproductive age an adequate folic acid to prevent spina bifida and anencephaly.

## Background

Congenital malformations are single or multiple defects of the morphogenesis of organs or bodies identified at birth or during the intrauterine life^1^. Congenital anomalies (CAs) could be minor or major malformation. Major structural malformations include anomalies in central nervous system anomalies (neural tube defects), the musculoskeletal system (orofacial clefts, and limb reduction defects), gastrointestinal tract, genitourinary system, and congenital heart disease (CHD). These malformations are life-threatening, having grievous effects on the well-being and survival of children born affected by these defects^1,2^.

The causes of the congenital anomalies could be genetic, environmental or a combination of factors^3–6^. Specifically, chemical toxicants, infection agents, maternal disease, and extracellular factors such as X-rays or chemical pollution, and periconceptional hormonal disruption are among the environmental factors that lead to congenital anomalies. Genetic factors are genetic chromosomal irregularities. Additionally, socioeconomic factors affect reproductive health by differentiating the exposure to the other risk factors as well as the access to prevention measures^5,6^.

Globally, it is estimated that 8 million babies that accounts about 6% of total births.

are born with a severe congenital birth defect^5–7^. March of Dimes global reports on CAs have shown that the hidden toll of mortality associated with CAs is estimated at 3.3 million children under age five years^2^. Most congenital malformations and almost all (95%) mortality due to the CAs among children occur in resource-poor settings^2,5–7^. Though, almost all birth defects and deaths due to the birth defects among children occur in low- and middle-income countries, there is still a paucity of sufficient reports on CAs^6^.

Recently, a hospital-based study on the incidence of neural tube defects (NTDs) has been done in the Tigray region, Northern Ethiopia^8^. Although the study was limited both in scope and type of anomalies studied. It has reported that the overall incidence rate of NTDs in the study was 131 per 10, 000 births of which 23% were live births and 77% were stillbirths. All the micronutrients are present in meat, fish, milk, fruits, and vegetables^9,10^. Most, if not all, Ethiopians rely on “injera” (a flat soft pancake like) made from cereals as the main source of food. Most of the poor in Ethiopia consume “injera” as 65% or more of their diet. Folate deficiency is widespread in Ethiopia and is related to diet. Prevalence of folate deficiency in Ethiopia, accounts for 46% and particularly in our study area, it accounts 54.4% of the population^11^, this further beefs up the condition observed in our study. Though the country has voluntary folic acid fortification policy, it needs to be legalized and mandatory to reduce the burden of the anomalies. The program that we have in Ethiopia is supplementation of iron foliates which is only prescribed for anemia suspected pregnant mothers. Apart from this, in Ethiopia, particularly in the Tigray regional state, there is currently a scarcity of birth defect data necessary to develop evidence-based prevention strategies. Therefore, this study was aimed to investigate the prevalence of congenital malformations in the Tigray region, Northern Ethiopia. And help to complement the ongoing scientific and policy dialogues for the intervention of the anomalies/NTDs in the region and the country at large.

## Methods

### Study design and period

Hospital-based cross-sectional study was conducted to identify neonates with major congenital anomalies in the labor ward admitted in six major public hospitals of Tigray region of Ethiopia between January 2018 and 2019.

### Study setting

Tigray Region is one of the nine regional state of Ethiopia, which has a total population of 4,316,988, of whom 2,126,465 are men and 2,190,523 women; urban residency for 19.55% of the total population. In the region, there are 992, 635 households, which accounts an average of 4.4 persons in a household, with urban households having an average 3.4 and rural households accounts 4.6 people^12^. The standard of living in Tigray region is distributed as 31.6% of the inhabitants fall into the lowest wealth quintile; adult literacy for men is 67.5% and for women 33.7%; and the regional infant mortality rate is 67 infant deaths per 1000 live births, which less than the nationwide average of 77; at least half of these deaths occurred in the infants' first month of life^13^.

Tigray has fourteen Hospitals, 170 Health Centers and 552 Health Posts (providers of basic health care and family planning in the rural areas). Of these fourteen Hospitals, six hospitals including Ayder Comprehensive Specialized Hospital, Lemelem Karl Hospital, St. Mary Hospital, Sihul Hospital, Adigrat Hospital, Alamata Hospital were selected randomly. These hospitals are the major public hospitals found in six administrative zones (Mekelle zone, Eastern zone, Central zone, North West zone, Southern zone, of Tigray region, serving the populations with diverse demographic characteristics as well as health-related behaviors.

According to The Ethiopian Demographic and Health Survey (EDHS) 2016, showed that Tigray region of Ethiopia has the recent 5 years coverage of ANC1 (antenatal care at least one visit) accounts 90%, ANC 4 + (at least four visits) in 57%, 5 years coverage of deliveries attended by skilled provider in 59%, and recent 2 years coverage of deliveries attended by skilled provider in 69%, and postnatal care was 47.7%, respectively. 57% of Tigray residents deliver at health facility or at one of the eight general hospitals^14^. While there is no clear data about where or how much other delivery occur (private hospitals, home births, etc.).

### Study population, sample size and techniques

All neonates delivered during the study period in Tigray region (for women who lived in Tigray region or in the catchment area for the 6 months prior to delivery) were included in this study. Cases were identified at delivery rooms soon after birth and were defined as live births, stillbirths, and/or terminations with any type of major congenital anomalies.

Induced abortions that have poor prenatal diagnosis of congenital anomalies in the governmental hospitals were not also included. Stillbirth is defined as the birth of an infant that has died in the womb (strictly, after having survived through at least the first 28 weeks of pregnancy).

### Study variables

#### Dependent variable

The dependent variable was major congenital malformation. The major congenital malformation were further classified as having only one major birth defect (isolated), having more than one major birth defect (multiple) including central nervous system anomalies, musculoskeletal system anomalies, musculoskeletal and neural tube defects, gastrointestinal tract anomalies, gastrointestinal tract and neural tube defects, genitourinary system, musculoskeletal and genitourinary defects, genitourinary and gastrointestinal defects, musculoskeletal and gastrointestinal defects, and conjoined twins.

#### Independent variables

The independent variables were gestational age, birth weight, sex, gravidity, parity, antenatal care, previous abortions, maternal illness, age, medication, and malformation history.

#### Operational definitions

- **Major structural anomalies:** are the conditions that account for most of the deaths, morbidity and disability related to CAs.
- **Multiple congenital anomalies:** Multiple congenital anomalies are the occurrence of two or more major anomalies that are unrelated. This means that the major anomalies are presumed to be a random association, and do not constitute a sequence or a previously recognized syndrome.
- **Isolated** congenital anomalies: major congenital anomalies that there are no other unrelated major congenital anomalies present. Frequently, major anomalies are associated with one or more minor anomalies.

### Data collection process

Data on child and maternal variables were collected in line with the labor ward records in each hospital. All relevant information (any type of major congenital malformations, demographic and obstetric factors) about neonates, mothers and the presence of major congenital defects detected within 24 h after delivery were collected. The required data were obtained from the gynecology and obstetrics (GYOB) ward in each hospital admission. Data were collected (retrieved) and recorded by 10 trained midwives, 10 midlevel obstetric professionals (trained in emergency surgery and obstetrics), 3 resident physicians and 6 senior obstetricians based on the availability of these professionals in the hospital. This means that we have at least one midwife and one emergency surgery obstetrics professional per shift in each hospital. We trained them for three consecutive days before the start of the study and later in the middle of the study we gave them and evaluated the data collection process for another three days as refreshment training. We standardized the questioner for all hospitals. We, the investigators, strictly supervised the data collection process in each hospital.

Every pregnant mother who delivered at gynecology and obstetrics (GYOB) ward were recorded. After delivery, all relevant information about neonates (gestational age, birth weight, sex, and presence of congenital malformations) was collected.

All malformations were classified as per the International Classification of Diseases (ICD) 10 classification. The assessment of gestational age was determined from the last normal menstrual period (LNMP) to the date of birth. This was best estimated using combinations of the LNMP, early clinical examination, and early ultrasound scans. Ultrasound examinations were not carried out routinely, because the equipment was always not available/ working at some of the hospitals. We, thus, confirm that all the data/experiment was performed accordance with relevant guidelines and regulations.

### Statistical analysis

The data collected was coded, cleaned, and analysed using Statistical Packages for Social Sciences (SPSS) version 20. The prevalence of congenital anomalies was calculated by dividing the total number of neonates with anomalies (live births, stillbirths, and/or terminations) delivered during the study period (Numerator) by the total number of live births, stillbirths, and/or terminations delivered at the study hospitals during the same time period (Denominator). The distribution of each congenital anomaly types was calculated by dividing the number of neonates with a specific anomaly by the total number of deliveries affected by congenital anomalies, multiplied by 100. Cross-tabulation (or chi-square analysis) was used to show the association between dependent and independent variables. The anomalies associated with maternal and fetal factors were assessed using the binary logistic regression, and the crude odds ratio, 95% confidence intervals and P-value were reported. The level of significance was set at *p* < 0.05.

## Results

A total of 12,225 births and terminations were observed in the six public hospitals over one year of the study period (Table 1). Three hundred eighty-three cases of major congenital malformation were observed during the study period. Overall prevalence of major congenital malformations was 3.13% of total births (Table 1).

**Table 1:** Hospital based prevalence of major congenital malformations in Tigray, Northern Ethiopia, 2019 (n = 12,225).

Hospital	Total births		Major congenital malformation			
	N	%	No congenital malformation	%	Yes (congenital malformation observed)	%
Ayder	3866	31.6	3700	30.2	166	1.4
Lemlem Karl	1195	9.7	1162	9.5	53	0.4
Alamata	1608	13.2	1573	12.8	35	0.29
Adwa	1317	10.7	1259	10.3	38	0.3
St Marry	2407	19.7	2348	19.2	59	0.48
Sihul	1832	15	1800	14.7	32	0.26
Total deliveries	12,225	100	11,842	96.7	383	3.13

Percentages were calculated by taking the corresponding “total number” as denominator.

### Typology of congenital anomalies

Congenital anomalies (CAs) of the central nervous system, specifically neural tube defects (NTDs) were the commonest anomalies in this study, found to be 68.7% (263NTDs/383 CAs) of the neonates with CAs (Table 2). The overall prevalence of NTDs was 2.15% (263/12,225 births) of the total births and terminations examined. Of the total NTDs cases identified, spinal bifida was the commonest NTDs type occurring in 60% (158/263NTDs cases), followed by anencephaly in 28% (73/263NTDs cases), encephalocele in 12% (32/263NTDs cases) and hydrocephalus 8.3% (32/383), respectively. Abdominal anomaly (omphalocele) in 18.2% (70/383 CAs), and club foot in 14.6% (56/383 CAs), were observed next to spinal bifida. Among the NTDs cases identified, more males have the defect than females (Table 3).

**Table 2:** Type and prevalence of the major congenital anomalies in Tigray, Northern Ethiopia, 2019.

Type of major congenital anomalies	Frequency	Percent major congenital anomalies per total births assessed	Percent of each type of major congenital malformations per total major congenital anomalies reported
Central nervous system anomalies	263	2.15	68.7
Musculoskeletal system anomalies	1	0.0	0.3
Gastrointestinal tract anomalies	11	0.1	2.9
Genitourinary system	12	0.1	3.1
Musculoskeletal and genitourinary defects	29	0.2	7.6
Genitourinary and gastrointestinal defects	29	0.2	7.6
Musculoskeletal and gastrointestinal defects	36	0.3	9.4
Conjoined twins	2	0.0	.5
Total	383	3.1	100.0
No congenital malformation	11,842	96.9	
Total	12,225	100.0	

Column 3, the percentage of neonate with congenital anomalies (CAs) was calculated as total number of CAs per total birth assessed (total CAs cases identified/total birth observed) and in column 4 the percentage of each type of CAs was calculated as identified type of CAs per total CAs observed (type of CAs identified /total CAs observed).

**Table 3:** Characteristics of the observed neural tube defects (n = 263).

Characteristics		Frequency	Percent (%)
NTDs type	Spina bifida	158	60
	Anencephaly	73	28
	Encephalocele	32	12
Associated anomalies with spina bifida	Hydrocephalous	32	8.3
	Club foot	56	14.6
	Omphalocele	70	18.2
Sex of neonate affected with NTDs (n = 263)	Male	153	58.2
	Female	110	41.8
Total	263	100	

Anomalies of musculoskeletal with gastrointestinal system identified in 9.4% (36/383CAs), genitourinary with gastrointestinal defects identified in 7.6% (29/383CAs), musculoskeletal with genitourinary defects identified in 7.6% (29/383CAs), genitourinary system observed in 3.1% (12/383CAs), and gastrointestinal tract anomalies occurring in 2.9% (11/383CAs) (Table 2). The congenitally malformed neonates (n = 383) identified in this study were classified into 10 categories according to the international classification of diseases version 10, ICD10 (Table 2).

### Association of major congenital malformation in relation to maternal and fetal characteristics

Table 4 has shown women 20 years of age and younger (OR = 1.882, 95% CI = 1.386–2.557, *p* < 0.0001) and women older than 35 years of age (OR = 2.905, 95% CI = 2.059–4.098, *p* < 0.0001), were more likely to have babies with congenital anomalies compared with women aged between 21 and 35 years. Similarly, abortion history (OR = 1.897, 95% CI = 1.460–2.465, *p* < 0.0001) and gravidity above 4 (OR = 1.634, 95% CI = 1.078–2.478, p = 0.005), were more likely associated with an increased risk of babies with congenital anomalies. Gravidity below 4, parity, pervious history of congenital anomalies, maternal illness, and medication history were less likely associated with an increased risk of babies with congenital anomalies.

**Table 4:** Association of major congenital malformation in relation to maternal and fetal characteristics, Tigray, Ethiopia.

Characteristics		Frequency	Odds ratio	95% confidence interval	P value
**Maternal characteristics**					
Maternal age	< = 20	1052	1.882	1.386–2.557	< 0.0001
	21–35(reference)	3285			
	> 35	540	2.905	2.059–4.098	< 0.0001
Abortion history	No (reference)	10,750	1.897	1.460–2.465	< 0.0001
	Yes	1475			
Gravidity	< = 2 (reference)	7592			
	3–4	2761	3.099	2.332–4.118	< 0.0001
	> 4	1872	1.782	1.160- 2.737	0.008
Parity	< = 2 (reference)	8701			
	3–4	2673	0.311	0.220–0.440	< 0.0001
	> 4	851	1.002	0.607–1.653	0.994
ANC	Yes (reference)	8653	0.909	0.714–1.157	0.439
	No	3572			
Previous history of congenital anomalies	Yes	295	0.771	0.354–1.676	0.511
	No (reference)	11,930			
Maternal illness	Yes	1047	1.127	0.775–1.638	0.533
	No (reference)	11,178			
Medication history	Yes	5556	0.948	0.750–1.198	0.654
	No (reference)	6669			
**Fetal characteristics**					
Sex	Male (reference)	5835			
	Female	6378	1.014	0.822–1.252	0.896
Gestational age	< = 28	696	2.058	1.437–2.948	< 0.0001
	29–31	1378	0.407	0.241–0.685	< 0.0001
	32–36	1895	0.548	0.371–0.809	0.002
		3370	0.406	0.284–0.581	< 0.0001
	37–38	39–40 (reference) 4596			
	> 40	290	3.686	2.410–5.637	< 0.0001
Birth weight	< = 2.5	3579	0.849	0.690–1.046	0.124
	> 2.5 (reference)	8166			

Gestational ages below 28 weeks (OR = 2.058, 95% CI = 1.437–2.948, *p* < 0.0001) and above 40 weeks (OR = 3.686, 95% CI = 2.410–5.637, *p* < 0.0001) were strongly associated with an increased risk of babies with congenital anomalies. Sex, birth weight, and gestational age (29-38 weeks) at birth were less likely associated with the occurrence of congenital anomaly.

### Ethics approval and consent to participate

The study was ethically approved by the Institutional Review Board of the College of Health Sciences, Mekelle University before the starting of the actual data collection. The possibilities of maltreatment or discomfort expected in this study were none existent. Well trained data collectors were used and strictly followed ethical principles. Verbal consent (as most of them were not able to read and write) was obtained from study participants before the beginning of the interviews and this was approved by the Health Research Ethics Review Committee (reference number: ERC 1279/2018). All study participants were assured that their participation was voluntary and if any discomfort, they could withdraw from the study without any problem at any stage of the data collection. Moreover, all data obtained from the respondent remained confidential and the dissemination of the study findings will be unspecified but the general source population.

### Consent for publication

Not Applicable.

## Discussion

This study has shown a high prevalence of major congenital malformations in the study community. The overall prevalence of congenital malformations was 3.13%. Our finding is higher than other studies conducted in: Egypt 2.5%^15^, Kolkata (India) 2.22%^16^, Odisha (India) 1.6% [117]. Central nervous system defect specifically NTDs were the common malformation identified in our study. This finding agrees with the findings from China^18^, Iran^19^ and Tanzania^20^ that reported CNS was the most common malformation observed in their study setting. On the contrary, musculoskeletal system defects were commonest in Egypt^15^, Kolkata (India)^16^, Odisha (India)^17^.

In the present study, a high number of NTDs were observed (2.15%) which is higher than the prevalence rate (1.31%) recently reported from the similar study setting (Tigray regional state of Ethiopian)^8^. Other study from Spain also reported 0.2% prevalence rate of NTDs^21^.

After randomized, controlled trials established that consumption of folic acid before pregnancy and during the early weeks of gestation reduces the risk of neural tube defect (NTD) in United States and Canada^22^. Assuming that the non-folic acid preventable NTDs rate should be 0.0005%^23^ and that our prevalence rate is 2.15%, then we have a rate/epidemic that is 43 times what it should be. This just screams urgency to implement effective/mandatory/ programs to get all women of reproductive age an adequate folic acid to prevent all folic acid related NTDs which could have prevented around 96% (2/2.15) of NTDs in the Tigray region.

In this study, more male neonates were affected with NTDs, in agreement with the previous study of the same study area^8^. Similar findings were reported from North African and Sub-Saharan countries as well^24,25^. However, the burden of NTDs was higher in female neonates in European reports^26,27^. The relation of the observed sex difference with the genesis of NTDs is not yet confirmed. This could indicate that different mechanisms are contributing to the genesis of NTDs and that require further investigation.

This study has shown that the maternal factors such as women 20 years of age and younger and women older than 35 years of age, abortion history, gravidity above 4, were more likely associated with an increased risk of babies with congenital anomalies. This finding is in line with findings reported from various countries, which showed maternal age, gravidity, abortion history and parity were significantly associated with incidence of major congenital malformations^15,28^.

Moreover, our study has shown fetal factors such as gestational ages below 28 weeks (*p* < 0.0001) and above 40 weeks (*p* < 0.0001) were strongly associated with an increased risk of babies with congenital anomalies. Similar findings have been reported from hospital-based studies conducted from different part of India^29,30^, and population-based study from China^18^.

As the study was not a community-based study, the prevalence could be artificially low because we only use data from a limited number of hospitals (not including other delivery locations) and not incorporating prevalence of anomalies after birth (i.e., many heart defects). Furthermore, the associated factors that were indicated in this study were not resulted from the multivariable logistic regression analysis. Hence, the result might be affected by possible confounding factors.

## Conclusion

This study has shown high prevalence of major congenital anomalies in the study community. Of the total congenital anomalies observed, most neonates are affected with neural tube defects, a birth defect with well–established evidence having folic acid deficiency or insufficiency is the predominate cause of spina bifida and anencephaly. This just screams urgency to implement effective/mandatory/ programs of folic acid supplementation to all women of reproductive age to prevent all of folic acid preventable NTDs in the Tigray Region.

### Data availability

All relevant data are within the paper.

## Abbreviations

CHD: Congenital heart disease
NTDs: Neural tube defects
CNS: Central nervous system
ICD10: International classification of diseases version 10
MSS: Musculoskeletal system
LMP: Last menstrual period
HIV: Human immunodeficiency virus
GUS: Genitourinary system
TCG: Thoracopagus
GIT: Gastrointestinal tract
GYOB: Gynecology and obstetrics

## References

[1] Corsello G, Giuffre M (2012). Congenital malformations. J. Maternal-Fetal. Neonatal. Med..

[2] Christianson A, Howson CP, Modell B. March of Dimes global report on birth defects, the hidden toll of dying and disabled children. White Plains, New York: March of Dimes Birth Defects Foundation, 2006.

[3] Castillo-Cadena J, Mejia-Sanchez F, Lopez-Arriaga JA (2017). Congenital malformations according to etiology in newborns from the floricultural zone of Mexico state. Environ. Sci. Pollut. Res. Int..

[4] Baldacci S, Gorini F, Santoro M, Pierini A, Minichilli F, Bianchi F. Environmental and individual exposure and the risk of congenital anomalies: a review of recent epidemiological evidence. *Epidemiol. Prev*. 2018 May-Aug;42(3–4 Suppl 1):1–34. 10.19191/EP18.3-4.S1.P001.057 30066535

[5] Moorthie S, Blencowe H, Darlison MW (2018). Estimating the birth prevalence and pregnancy outcomes of congenital malformations worldwide. J. Commun. Genet..

[6] Ajao AE, Adeoye IA (2019). Prevalence, risk factors and outcome of congenital anomalies among neonatal admissions in OGBOMOSO Nigeria. BMC Pediatr.

[7] 10.1186/s12887-019-1471-1

[8] Berihu BA, Welderufael AL, Berhe Y, Magana T, Mulugeta A, Asfaw S (2018). High burden of neural tube defects in Tigray, Northern Ethiopia: Hospital-based study. PLoS ONE.

[9] Zaganjor I, Sekkarie A, Tsang BL, Williams J, Razzaghi H, Mulinare J, et al. Describing the prevalence of neural tube defects worldwide: a systematic literature review. *PLoS One* 2016; 11:e151586. 10.1371/journal.pone.0151586 PMC4827875 27064786

[10] Safi J, Joyeux L, Chalouhi GE. Periconceptional folate deficiency and implications in neural tube defects. *J. Pregnancy* 2012; 2012:295083. 10.1155/2012/295083 PMC3415073 22900183

[11] Haidar J, Melaku U, Pobocik RS (2010). Folate deficiency in women of reproductive ages in nine administrative regions of Ethiopia; an emerging public health problem. South Afr. J. Clin. Nutrit..

[12] bālaśelṭān EYs, Macro O. Ethiopia Demographic and Health Survey, 2005: Central Statistical Authority 2006.

[13] Demographic E. Health survey 2011 central statistical agency Addis Ababa. Ethiopia ICF International Calverton, Maryland, USA 2012.

[14] Central Statistical Agency (CSA) [Ethiopia] and ICF. 2016. Ethiopia Demographic and Health Survey 2016: Key Indicators Report. Addis Ababa, Ethiopia, and Rockville, Maryland, USA. CSA and ICF.

[15] El Koumi MA, Al Banna EA, Lebda I. Pattern of congenital anomalies in newborn: a hospital-based study. Pediatr Rep. 2013;5:e5. 10.4081/pr.2013.e5 PMC3649744 23667734

[16] Sarkar S, Patra C, Dasgupta MK, Nayek K, Karmakar PR (2013). Prevalence of congenital anomalies in neonates and associated risk factors in a tertiary care hospital in eastern India. J. Clin. Neonatol..

[17] Agrawal, D., Mohanty, B.B., Sarangi, R., Kumar, S., Mahapatra, S.K., Chinara, P.K. Study of incidence and prevalence of musculoskeletal anomalies in a tertiary care hospital of eastern India. J. Clin. Diagn. Res. 2014;8:AC04–6. 10.7860/JCDR/2014/7882.4380 PMC4079988 24995167

[18] Zhang X, Li S, Wu S, Hao X, Guo S, Suzuki K (2012). Prevalence of birth defects and risk-factor analysis from a population-based survey in Inner Mongolia China. BMC Pediatr..

[19] Golalipour MJ, Marfazeli A, Mobasheri E (2013). Incidence and pattern of congenital malformation in Gorgan-North of Iran. J. Med. Sci..

[20] Mashuda F, Zuechner A, Chalya PL, Kidenya BR, Manyama M (2014). Pattern and factors associated with congenital anomalies among young infants admitted at Bugando medical centre, Mwanza. Tanzania. BMC Res Notes..

[21] Martinez-Frias M, Cereijo A, Bermejo Eea: Epidemiological aspects of Mendelian syndromes in a Spanish Population sample I. Autosomal dominant malformation syndromes. *Am. J. Med. Genet.* 1991, 38:622–625. 10.1002/ajmg.1320380424 2063907

[22] Berry RJ, Bailey L, Mulinare J, Bower C, Dary O (2010). Fortification of flour with folic acid. Food Nutrit Bull.

[23] Arth A, Kancherla V, Pachón H, Zimmerman S, Johnson Q, Oakley GP (2016). Status of global prevention of folic acid preventable-spina bifida and anencephaly. Birth Defects Res A Clin Mol Teratol..

[24] UNICEF: Child info-UNICEF-Monitoring the situation of children and women. In.; 2005.

[25] Adeloye-Blantyre-Malawi A. Spina bifida cystica in the African*. Afr. J. Neurol. Sci.* 1995;14(2).

[26] Mabogunje OA (1990). Spina bifida cystica in northern Nigeria. Childs Nerv. Syst..

[27] Juriloff DM, Harris MJ (2012). A consideration of the evidence that genetic defects in planar cell polarity contribute to the etiology of human neural tube defects. Birth Defects Res. A Clin. Mol. Teratol..

[28] Juriloff DM, Harris MJ (2012). Hypothesis: the female excess in cranial neural tube defects reflects an epigenetic drag of the inactivating X chromosome on the molecular mechanisms of neural fold elevation. Birth Defects Res. A Clin. Mol. Teratol..

[29] Taksande A, Vilhekar K, Chaturvedi P, Jain M (2010). Congenital malformations at birth in Central India: a rural medical college hospital based data. Indian J. Hum. Genet..

[30] Neelambari YC (2018). Prevalence, pattern and outcome of congenital malformations in a tertiary care centre in South India. Int. J. Contemp. Pediatr..

